# The Relationship between Parasite Fitness and Host Condition in an Insect - Virus System

**DOI:** 10.1371/journal.pone.0106401

**Published:** 2014-09-10

**Authors:** Michelle Tseng, Judith H. Myers

**Affiliations:** Department of Zoology, University of British Columbia, Vancouver, British Columbia, Canada; George Washington University School of Medicine and Health Sciences, United States of America

## Abstract

Research in host-parasite evolutionary ecology has demonstrated that environmental variation plays a large role in mediating the outcome of parasite infection. For example, crowding or low food availability can reduce host condition and make them more vulnerable to parasite infection. This observation that poor-condition hosts often suffer more from parasite infection compared to healthy hosts has led to the assumption that parasite productivity is higher in poor-condition hosts. However, the ubiquity of this negative relationship between host condition and parasite fitness is unknown. Moreover, examining the effect of environmental variation on parasite fitness has been largely overlooked in the host-parasite literature. Here we investigate the relationship between parasite fitness and host condition by using a laboratory experiment with the cabbage looper *Trichoplusia ni* and its viral pathogen, AcMNPV, and by surveying published host-parasite literature. Our experiments demonstrated that virus productivity was positively correlated with host food availability and the literature survey revealed both positive and negative relationships between host condition and parasite fitness. Together these data demonstrate that contrary to previous assumptions, parasite fitness can be positively or negatively correlated with host fitness. We discuss the significance of these findings for host-parasite population biology.

## Introduction

Parasites play a significant role in the ecology and evolution of their hosts. For example, parasites can regulate host population dynamics [1]–[3], drive the maintenance of host sexual reproduction [4]–[6], and shape the evolution of sexually dimorphic traits [7]. Environmental variation can play a large role in mediating the immediate outcome of parasite infection, as hosts that are reared in crowded conditions or with limited food can suffer greater morbidity or mortality from parasitism compared to hosts in better health [8]–[14]. Far less is known about how stressful conditions for the host such as crowding or food limitation affect the fitness of the parasites. Examining this question is a subtle but significant departure from most host-parasite studies, where the focus is primarily on host performance. Understanding how environmental factors affect parasite fitness might result in more accurate predictions regarding the number of parasite propagules available for subsequent infection. This information can in turn result in more accurate predictions regarding both the likelihood of infection, and the severity of infection.

How might variation in the host’s environment affect parasite fitness? For parasites that depend solely on their hosts for resources and shelter, a poor environment for the host may translate into a poor environment for the parasite. For example, parasites inhabiting low-quality hosts may have less to eat (both quantitatively and qualitatively), which may reduce parasite production [15], [16]. Conversely, hosts in poor condition may have fewer resources to allocate to immune functions or to other defenses against parasites [17], [18] thus leaving parasite growth and or reproduction less inhibited by attack from host defenses.

As a sidebar, we note here that in general, lifetime parasite fitness is typically defined as the parasite basic reproductive ratio, R_0_, but because of the multiple components that make up R_0_ [19]–[22], many studies instead use parasite productivity as a measure of parasite fitness (e.g. [16], [22]–[24] but see [25] for measures of lifetime parasite fitness). Parasite productivity is a reasonable proxy for R_0_, if productivity is correlated with the number of transmission propagules produced, and if the latter is positively correlated with the likelihood of infecting a susceptible host (e.g. [26], [27]).

Here we use the term ‘potential parasite fitness’ (PPF) because we do not directly measure parasite R_0_. Rather, we measure components of parasite fitness that are typically positively correlated with R_0_. In this study we ask whether parasite productivity is positively correlated with host food availability (a proxy of host condition) in the virus *Autographa californica* multiple nucleopolyhedrovirus (AcMNPV), and one of its natural hosts, the cabbage looper moth (*Trichoplusia ni*, Hübner, Lepidoptera: Noctuidae).

## Methods

### Parasite biology

AcMNPV is the type species of the genus *Alphabaculovirus* in the family *Baculoviridae* [28]. Baculoviruses are DNA viruses that primarily infect Lepidoptera [29], [30]. Caterpillars typically become infected upon ingesting virus occlusion bodies (OB), which are proteinaceous structures that contain virions (virus particles) [30]. Virions released by OBs spread throughout the larval body, and eventually the bulk of host tissue is converted into OBs [30]–[32]. At the end of a successful infection, the larva dies and OBs are released into the environment. AcMNPV has a wide host range and can infect species of at least 15 families of Lepidoptera [30].

### Host biology

*Trichoplusia ni* are typically found in the subtropics worldwide [33] and are also common pests of greenhouse vegetables and agricultural cole crops at higher latitudes [34]. AcMNPV has been considered as a possible biological control agent of *T. ni* [35], [36] because it infects *T. ni* in the wild and has high virulence. This virus-host system is thus ideal for addressing questions related to PPF because the laboratory results could be applicable in nature, as well as to other lepidopteran hosts.

### Insect collections and colony maintenance

Cabbage loopers were collected from commercial greenhouses in the lower mainland of British Columbia, Canada and maintained continuously in the laboratory at the University of British Columbia for 10 years (∼50 generations). AcMNPV was originally isolated from naturally infected *T. ni* early 2000s. The virus was used in various laboratory experiments and was purified and stored at −20°C when not in use [37].

To maintain *T. ni* colonies, neonates were reared in groups of 25 in 200 mL Styrofoam cups filled with 25 mL wheat-germ based diet [38]. Pupae were transferred to in emergence cages. Adults mated in these cages and females laid their eggs on a paper towel lining of the mating cage. Larval rearing cups and adult mating cages were maintained at 26±1°C 16∶8 light:dark. Egg-impregnated paper towels were stored at 5° until eggs were needed. *Trichoplusia ni* eggs readily hatch at room temperature.

### Experimental design

We conducted two experiments to examine the relationship between parasite potential fitness and host condition in this host-parasite system. In both experiments we infected 4^th^ instar larvae with virus, but the larvae used in experiment 1 were of lower initial condition than those used in experiment 2. We conducted these two experiments to gain a preliminary understanding in how host condition at the time of infection affects both host and parasite overall response to infection. Table 1 lists the differences between the two experiments. The experiments were conducted at two different times because of logistical constraints.

**Table 1:** Experimental design for experiments 1 and 2.

	Experiment 1	Experiment 2
Reared in group-rearing cups:	1^st^ to 4^th^ instar	1^st^ to 3^rd^ instar
Transferred to individual rearing cups:	4^th^ instar	3^rd^ instar
Assigned to food treatments (low, medium, high):	4^th^ instar	3^rd^ instar
Infected with virus:	4^th^ instar	4^th^ instar
Expected host condition at time of infection:	Lower	Higher

For each experiment, 120 larvae were each assigned to one of three food regimes: low (4–5 hours access to food/day), medium (12 hours food/day), or high (continuous access to food). Larvae were reared in 25 mL cups and the food source was a wheat germ-based diet modified from [38]. Larvae in experiment 1 were assigned to their food treatment after infection and larvae in experiment 2 were assigned to their food treatment before infection (Table 1).

One day after moulting into 4^th^ instar, 90 of the 120 larvae were each given one 0.125 cm^3^ piece of diet dosed with 5 µL of 1000 OB/µL virus suspension. Preliminary data have shown this infection method to be sufficient to infect ≥95% of larvae. After 24-hour access to the virus-dosed diet, larvae were assigned to their food treatment (experiment 1), or returned to their initial food treatment (experiment 2). The remaining thirty larvae were each fed a 0.125 cm^3^ piece of diet dosed with 5 µL distilled water. These uninfected larvae were randomly and evenly distributed into the three food treatments (i.e. 10 uninfected larvae per food treatment, experiment 1), or returned to their original food treatment (experiment 2).

### Data collected and statistical analyses

Infected larvae were maintained on low, medium or high food treatments until death. One day prior to death, when larvae were rendered immobile, bloated and discoloured by virus infection, larvae were weighed and transferred to 1.5 mL eppendorf tubes. After death, the tube containing the virus-killed larva was filled with distilled water so that the total volume (dead larva plus water) equaled 1 mL. The entire sample was macerated and total OB number was quantified using a hemocytometer. Virus OBs were counted in each of ten 0.2×0.2 mm squares. The average number of OBs per ten squares was then multiplied by 4×10^6^ to obtain the total OB per larva. We collected data on days to death, weight at death, and total OBs per larva. We use virus OB number as our measure of PPF.

We used ANOVA to examine whether food treatment had a statistically significant effect on larval weight at death, days to death, and on virus OB number. We transformed both OB number (log OB number +1) and larval weight (log-weight) to meet assumptions of ANOVA. To better understand the functional relationship between virus productivity and host size, we used ANCOVA to examine whether food treatment mediated the relationship between OB number and larval weight (dependent variable: log OB+1), explanatory variable: food treatment, covariate: log (weight at death); interaction: food * log(weight at death). All statistical analyses were conducted in R version 3.0.2 (R Core Team 2013). Because the two experiments were conducted at different times and thus larvae could have been exposed to different environmental conditions in the laboratory, all statistics were run separately for the two experiments.

### Literature survey

We used the Web of Science to search for papers that experimentally addressed whether parasite fitness (e.g. parasite growth rate, reproduction, development, transmission potential) was affected by host condition (e.g. food quality or quantity). We did not include parasitoids in our search. We examined correlational or observational studies between host quality and potential parasite fitness separately from experimental papers. We do not claim to have found all relevant published studies: our goal was primarily to develop a broad understanding of whether general patterns exist between host and parasite fitness.

## Results

### Infection rate and overall sample size

None of the control, uninfected cabbage loopers died of viral infection. Because the goal of this study was to examine the effect of host food levels on parasite fitness, these uninfected control larvae were not included in statistical analyses. Larvae that did not develop full viral infections were also not included in the analysis. In experiment 1, three larvae that had been dosed with virus did not produce any virus OBs (1 larva from the low food treatment; 2 from the medium food treatment). Also for one or two larvae from each food treatment we missed the death date, or were unable to collect weight data because the larva had burst before its intact post-death weight could be recorded. Across the three food levels, data for days to death were collected from 81 larvae, and data for OB number and weight at death were collected from 83 larvae.

In experiment 2, no virus OBs were produced in 18 larvae (n = 13, 1, 4, in the low, medium and high food treatments respectively). Because almost half of the larvae (13/30) in the low food treatment did not develop a typical virus infection, we took a closer look at these unsuccessful infections. It appears that not only did these larvae not develop a proper virus infection, but they also barely grew at all. Unsuccessfully infected larvae died at a much lighter weight than infected larvae (F_1,26_ = 13.78, = 0.001), but there was no difference in days to death (Kruskal-Wallis χ^2^ = 1.78, p = 0.18). It is unclear why so many of the larvae in the low food treatment did not become infected with virus or why they did not grow in general. Overall, the infection rate was 57%, 97% and 87% for the low, medium and high food treatments respectively.

In addition, two infected larvae from the low food treatment burst before they were weighed, so we could not collect weight data for those individuals. Thus, across the three food levels, data for OB number and days to death were collected from 72 larvae, and weight at death were collected from 70 larvae.

### Host weight at death, time to death, and virus OB production

**Figure 1:**
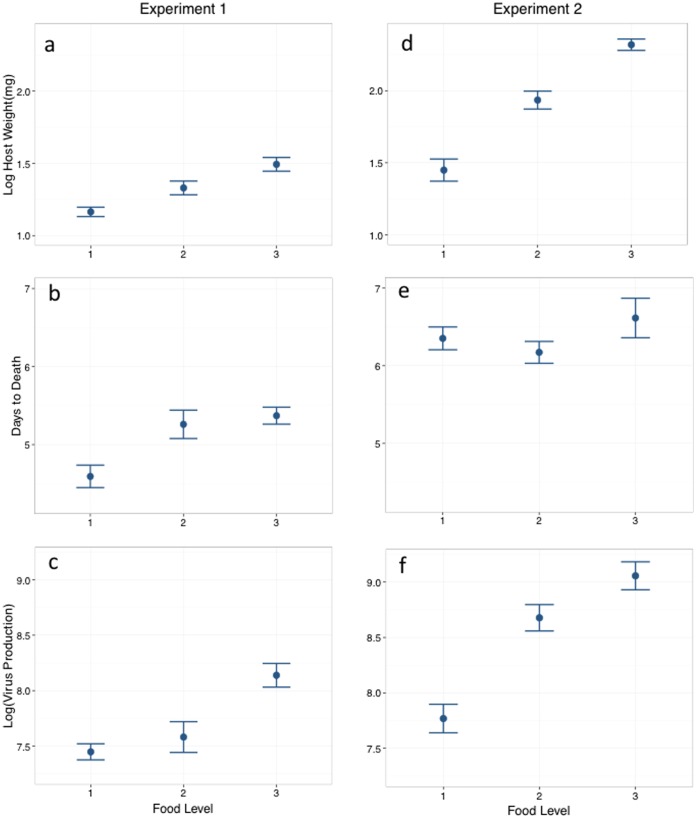
The effect of food treatment on host weight, host days to death, and total virus OB production for experiment 1 (a–c) and experiment 2 (d–f). Virus OB units are Log(OB per larva +1). Error bars are +/−1 S.E.M. Food treatments: 1 = low, 2 = medium, 3 = high. See Table 2 for ANOVA results.

Host weight at death and virus OB production increased with increasing food availability in both experiments (Fig. 1a, d; Fig. 1c, f; Table 2). In experiment 1, larvae that were fed medium and high food lived longer than those given low food (Fig. 1b; Table 2). Food treatment did not affect days to death in experiment 2 (Fig. 1e; Table 2).

**Table 2:** a. ANOVA table showing a significant effect of host food level on host weight, host days to death and virus production in Experiment 1, and on host weight and virus production in Experiment 2.

a. Analysis of variance results			
Factor	Dependent variable	Experiment 1	Experiment 2
Host food level	log(host weight)	F_2,80_ = 14.83; p<0.001	F_2,67_ = 45.2; p<0.001
	host days to death	F_2,78_ = 8.10; p<0.001	F_2,69_ = 1.45; p = 0.24
	virus production (log(OB+1))	F_2,80_ = 11.48; p<0.001	F_2,69_ = 22.7; p<0.001
**b. Analysis of covariance results**			
Factor*covariate	**Dependent variable**	**Experiment 1**	**Experiment 2**
Host food level*log(host weight)	log(virus OB+1)	F_2,77_ = 3.21; p = 0.046	F_2,64_ = 6.51; p = 0.003
**c. Slopes and p values for the relationship between log(virus OB+1) and log(host weight), per food treatment**			
		**Experiment 1**	**Experiment 2**
Food treatment	Low	1.57; p<0.001	0.13; p = 0.76
	Medium	2.56; p<0.001	1.74; p<0.001
	High	1.95; p<0.001	1.70; p<0.01

b. ANCOVA results showing that the relationship between virus production and host weight was not equal among the three food levels in both Experiment 1 and 2. c. Table of slopes and p-values for the relationship between OB production and host weight, per food treatment.

### Relationship between virus production, host weight and food treatment

**Figure 2:**
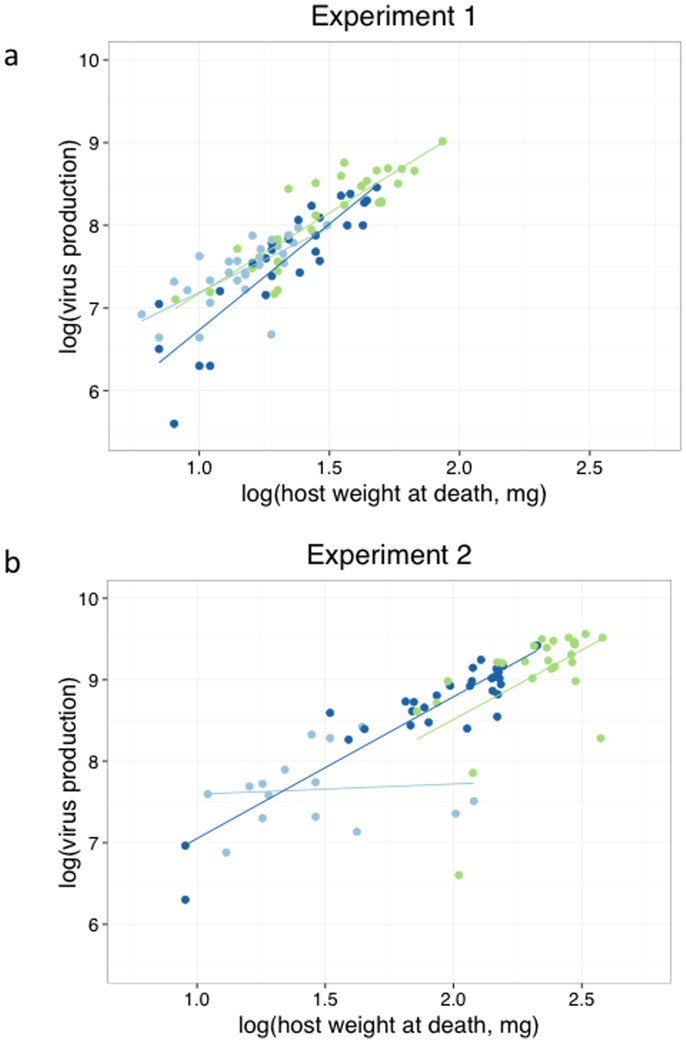
The relationship between virus productivity and host weight depends on host food treatment (a: experiment 1, b: experiment 2). Slopes of the relationships are shown in light blue, dark blue and green for the low, medium and high food treatments respectively. See Table 2 for ANCOVA results and for slope values.

We examined the slope of the relationship between virus production and host weight for each food treatment. In both experiments 1 and 2, the slope of this relationship was shallowest for the low food treatment (Fig. 2a, b; Table 2).

### Literature survey

We found 21 studies that demonstrated an increase in PPF with increasing host condition. We also found five studies where PPF decreased with increasing host condition, seven studies where PPF increased or decreased with host condition (depending on the parasite trait), and one study in which there was no change in parasite potential fitness (Table 3). Of these 34 papers, 17 documented host-parasite interactions in invertebrate hosts, 12 in vertebrate hosts, four in plant hosts and one paper investigated parasites of protists.

**Table 3:** Summary of experiments examining the effect of host food availability or food quality on fitness-related parasite traits.

	Parasite	Parasite Type	Host	Host Type	Host Treatment	Parasite Trait Measured	Trait +/− with + hostcondition	Reference
1	*Coccipolipus hippodamiae*	Mite	*Adalia pipunctata*	Insect	Food level	Egg number, development time	+	[61]
2	*Vavraia culicis*	Microsporidian	*Aedes aegypti*	Insect	Food level	Spore production	+	[24]
3	*Ascogregarina culicis, Vavraia*	Protozoan,Microsporidian	*Aedes aegypti*	Insect	Food level	Ascogregarina oocyst number	+	[62]
4	*Ascogregarina taiwanensis*	Protozoan	*Aedes albopictus*	Insect	Food level	Ooocyst number	+	[63]
5	*Crithidia bombi*	Trypanosome	*Bombus terrestris*	Insect	Food level	Transmission cells	+/−	[64]
6	*NPV*	Virus	*Operophtera brumata*	Insect	Food quality	Yield	+	[51]
7	*Hymenolepis diminuta*	Cestode	*Tribolium confusum*	Insect	Food level	Size	+	[65]
8	*Metschnikowia bicuspidate*	Fungus	*Daphnia dentifera*	Crustacean	Food level	Spore number, survival	+	[66]
9	*Metschnikowia bicuspidate*	Fungus	*Daphnia dentifera*	Crustacean	Food quality	Size, spore number	+/−	[67]
10	*Pasteuria ramose*	Bacteria	*Daphnia magna*	Crustacean	Food level	Spore number	+	[68]
11	*Pasteuria ramosa*	Bacteria	*Daphnia magna*	Crustacean	Food level	Offspring number	+	[23]
12	*Pasteuria ramosa*	Bacteria	*Daphnia magna*	Crustacean	Food level	Spore number	+	[69]
13	*Pasteuria ramosa*	Bacteria	*Daphnia magna*	Crustacean	Food level	Spore number	−	[70]
14	*Pasteuria ramose*	Bacteria	*Daphnia magna*	Crustacean	Food level	Growth	+	[71]
15	*Schistocephalus solidus*	Tapeworm	*Macrocyclops albidus*	Copepod	Food level	Growth	+	[72]
16	*Diplostomum spathaeceum*	Trematode	*Lymnaea stagnalis*	Snail	Food level	Transmission stage	+	[16]
17	*Microphallus*	Trematode	*Potomopyrgus antipodarum*	Snail	Food level	Development	+	[73]
18	*Amoebophrya sp*	Dinoflagellate	*Gymnodinium sanguineum*	Protist	Food quality	Spore number	+	[74]
19	*Arceuthobium vaginatum subsp.* *Cryptopodum*	Plant	*Pinus ponderosa*	Plant	Host density	Growth	+	[43]
20	*Podosphaera plantaginis*	Fungus	*Plantago lanceolata*	Plant	Food quality	Growth	+/−	[10]
21	*Botrytis cinerea*	Fungus	*Solanum lycopersicum*	Plant	Nutrient level	Growth	+	[75]
22	*Eurosta solidaginis*	Gall making fly	*Solidago altissima*	Plant	Nutrient level	Larval mass	No effect	[76]
23	*Crataerina melbae*	Flea	*Apus melba*	Bird	Food level	Survival	+/−	[77]
24	*Molothrus ater*	Bird (cowbird)	*Melospiza melodia*	Bird	Food level	Fledgling number	+	[78]
25	*Ceratophyllus gallinae*	Flea	*Parus major*	Bird	Host density	Offspring survival	+	[79]
26	*Ceratophyllus gallinae*	Flea	*Parus major*	Bird	Food level	Egg number	+/−	[80]
27	*5 species of nematodes*	Nematode	*Lepus americanus*	Hare (snowshoe)	Food level	Abundance	+/−	[81]
28	*Plasmodium falciparum*	Protozoan	*Homo sapien*	Human	Host quality	Infectivity	+	[82]
29	*Schistosoma japonicum*	Nematode	*Sus domesticus*	Pig	Food quality	Infectivity	−	[83]
30	*Trichuris suis*	Nematode	*Sus domesticus*	Pig	Food quality	Abundance	−	[12]
31	*Elaphostrongylus cervi*	Nematode	*Cervus elaphus*	Red deer	Food level	Abundance	−	[84]
32	*Strongyloides ratti, Nippostrongylus* *brasiliensis*	Nematode	*Rattus norvegicus*	Rodent	Immunecompromised	Development	+/−	[85]
33	*Xenopsylla ramesis*	Flea	*Meriones crassus*	Rodent	Food level	Survival	−	[86]
34	*Echinostoma caproni*	Trematode	*Mus musculus*	Rodent	Food quality	Abundance, size	+	[87]

(+ indicates that parasite fitness increased with host food or condition; − indicates a decrease in parasite fitness with host food or condition; +/− indicates that some parasite fitness traits increased and some decreased.

The types of parasites investigated included virus, bacteria, fungi, tapeworms, trematodes, nematodes, protozoans, insects, cowbirds and mistletoes. The parasite fitness traits quantified included: production of reproductive bodies (spores, oocysts) or transmission stages, growth, development time, abundance and survival (Table 2).

Although they were not included in Table 3, we also found seven studies that used correlational or observational studies to examine the relationship between parasite fitness traits and host condition. These papers included parasite fitness data for vertebrate hosts (stickleback/cestodes [15], voles/trypanosomes [39], doves/lice [40], rodent/cestode [41]; small mammals/protozoans and helminthes: [42]), and in a plant-mistletoe host-parasite system [43], [44]. The results of these correlational studies showed no clear relationships between parasite fitness and host condition.

## Discussion

### Empirical results

Our experiments revealed a strong positive relationship between virus productivity and host food availability. These results suggest that virus potential fitness likely benefits from increased resource availability to hosts in this host-parasite system.

We have also shown that the rate of virus OB production was lowest in hosts given the lowest access to food (Fig. 2a, b; Table 2). This result implies that in poorly-fed hosts, the virus is less efficient at converting host tissue to virus tissue. It is unclear why this might be the case; perhaps a stressed, low-condition larva translates into a low-quality or low-quantity resource for the virus. In a laboratory experiment with western tent caterpillar, low food availability appeared to reduce the susceptibility of western tent caterpillars to NPV infection [45]. The authors suggested that this result was related to the immune function of the host when food deprived, or the ability of the virus to replicate for some other reason.

The overall positive relationship between virus productivity and host food availability across the three food treatments could also be linked to the relationship between larval mass and larval volume; in other words, virus OB production may be constrained by the volume of the insect. Previous literature [46] examined the relationship of larval weight to volume in *Heliconius cydno* (Lepidoptera: Nymphalidae) and *Trirhabda germinata* (Coleoptera: Chyrsomelidae) and found it to be linear on a log-log scale. The slope of the relationship was 1.03 for *T. germinata* and 0.95 for *H. cydno*. Overall, this linear log-log relationship is similar to the pattern observed in our experiments, and may suggest that virus production may be bounded by the rate at which larval volume increases with larval mass.

We conducted two experiments with slightly different starting conditions in order to gain a preliminary understanding of how host condition at the time of infection might affect overall host condition and parasite fitness. Because of logistical constraints, the two experiments were conducted at different times, so we make comparisons between the two experiments with some caution. Data for final host weight suggest that larvae in experiment 1 were had lower overall condition than those in experiment 2 (Fig. 1a, d). In fact the final weight of larvae in the lowest food treatment in experiment 2 overlapped with the final weight of larvae in the highest food treatment in experiment 1. Thus, transferring larvae from group rearing cups to individual cups at the third instar stage had a considerable affect on the overall size of the larvae, and on overall virus production. Unfortunately because of the small time-window available for larval infections at the early 4^th^ instar stage, we did not collect data on initial larval weight, so we do not have data on how much growth took place during the experiments. However, we still feel confident in the overall conclusion that increases in host condition are beneficial to the PPF in this host-parasite system.

With respect to ‘days to death’, infected larvae that had greater access to food also lived longer in Experiment 1, but in Experiment 2 food availability did not affect survival. The range of days to death observed here (4.5∼6.5 days; experiments combined), is within the range documented by other experiments with *T. ni* and AcMNPV [47]–[49], and the results for experiment 2 approach the upper end of those seen in comparable studies. The data suggest that across the two experiments hosts that were fed more also took longer to die, but again we say this with caution because the experiments were conducted at two different times.

Although we were unable to find published studies that similarly experimentally examined virus yield in response to host food availability, other authors have demonstrated that virus OB production was influenced by the type of plant fed to the host [50], [51]. Virus yield was highest in larvae of the western tent caterpillar (*Malacosoma californicum pluviale*) that were fed alder plants, versus wild rose or apple [50]. Similarly, virus yield was highest when winter moth (*Operopthera brumata*) larvae were fed oak, versus Sitka spruce or heather [51]. Together these data suggest that viral fitness is potentially affected by both larval food quantity and quality.

### Literature survey

The literature survey revealed that potential parasite fitness typically increased with host food availability in invertebrate, plant and protist hosts (Table 3). Our empirical data are consistent with these results.

For vertebrate hosts, the relationship between potential parasite fitness and host condition was more variable and tended to depend on the host-parasite system or on the parasite trait measured.

Overall, data from studies included in this literature survey suggest that PPF can both increase and decrease with host condition. We caution that this is a preliminary survey of the literature and a more comprehensive literature review or meta-analysis, which corrects for phylogenetic biases and variation in sample sizes are required to discuss any trends with quantitative rigor.

### Implications for understanding parasite fitness and disease ecology

Previous work in this area [13], [52] postulated that low condition hosts can be both more susceptible to parasites, and can suffer more from infection, than hosts in better condition. This increased susceptibility and suffering lead to what the authors termed a ‘vicious circle’, in which poor condition leads to higher parasite loads, which in turn keeps the host in poor condition. These ‘vicious circles’ can lead to individual reproductive failure and death, as well as host population decline [13]. Our empirical data and preliminary literature survey have demonstrated that poor condition hosts may have lower parasite productivity than higher condition hosts; thus in some host-parasite combinations, the ‘vicious circle’ may not lead to a continuous increase in parasite propagule pressure. Poor condition hosts may still suffer more from parasites than hosts in better condition, but they may end up contributing fewer parasites to the overall parasite population pool. If environmental conditions are poor across a large landscape (e.g. widespread drought), this may result in large numbers of poor-condition hosts, and for some taxa, in a decrease in parasite population size, rather than an increase.

We propose that the next step to a better understanding the relationship between host and parasite fitness is to examine in greater detail whether groups of taxa exhibit similarities with respect to the effect of environmentally-mediated variation in host condition on parasite fitness. Analytical or simulation models can then incorporate these general patterns to make predictions regarding the overall effect of variation in host condition on host-parasite dynamics (e.g. Daphnia/bacteria: [53]; Lepidopteran/virus: [26], [54], [55].

Given that the world is a heterogeneous place, environmentally-mediated variation in host condition is likely to be ubiquitous in nature. However, the outcome of parasite infection is not only affected by food availability or quality, as documented here, but also by factors such as variation in temperature [56]–[59] and salinity [60]. The goal moving forward is to determine whether broad patterns exist in how hosts and parasites respond to variation in these biotic and abiotic factors, and to use these patterns to inform how environmental heterogeneity affects host-parasite interactions at the population and community levels.
